# Effects of goal orientation on environment management in technology-based physics learning

**DOI:** 10.3389/fpsyg.2022.1048143

**Published:** 2023-01-09

**Authors:** Yanchao Yang, Jianxia Du, Timothy Teo, Sijia Xue, Fangtong Liu

**Affiliations:** ^1^Qinggong College, North China University of Science and Technology, Tangshan, China; ^2^Faculty of Education, University of Macau, Taipa, Macau SAR, China; ^3^Department of Educational Psychology, Faculty of Education, The Chinese University of Hong Kong, Shatin, Hong Kong SAR, China; ^4^College of Teacher Education, Southwest University, Chongqing, China; ^5^School of Foreign Languages, Shanxi University, Taiyuan, China

**Keywords:** environment management, goal orientation, time management, technology-based physics learning, physics learning

## Abstract

The purpose of the current study is to propose and examine a comprehensive model that uses motivational and self-regulated variables to explain factors affecting environment management in technology-based physics learning among Chinese secondary school students. Data were collected from 726 grade-eight secondary school students in Southeast China, who were learning physics. Structural equation modeling was used to analyze the relationships among students’ goal orientations, environment management, and time management. Results suggest that students were more likely to manage their environment if they had learning-oriented goals and if they managed their time, but they were less likely to do so if they had social-oriented goals. Implications for teachers’ technology integration in physics class were discussed.

## 1. Introduction

The management of studying environment, as an important strategy of self-regulation and volitional control, is crucial to completing learning tasks and realizing learning goals (Zimmerman and Pons, 1986; Zimmerman and Martinez-Pons, 1990; Corno, 1993; Wolters, 1998; Zimmerman, 1998a; Pintrich, 2000b). However, environment management is inherently challenging because external contextual factors such as physical settings and peers often go beyond a learner’s control (Pintrich, 2000b, 2004). Environment management in learning physics can be more challenging because physics is often perceived by learners, especially secondary school students, to be difficult, irrelevant and labor-intensive (Williams et al., 2003; Angell et al., 2004; Barmby and Defty, 2006). Due to the high cognitive demand and lack of interest in learning physics, learners could become distracted more easily if they do not have useful self-regulation skills, especially environment management strategies.

Many teachers use technology to improve learners’ experiences in physics learning, as a result of the increasing technology integration trend in education (Beichner et al., 1999; Jönsson, 2005). For instance, This study by Zhai and Shi (2020) tracked 803 high school students for 5 months as they used 15 mobile functions for physics learning. Pedagogical roles played by students and teachers significantly moderated the relationship between students’ perception of mobile functions and their achievement in physics. More importantly, it found that mobile use frequency mediated the relationship between students’ perception of mobile functions and their physics learning outcomes. Similarly, in his study, Zhai et al. (2018) examined how high school students used mobile devices in physics classrooms and after school, and investigated how their use of mobile devices affected their performance and interest in physics. Mobile technology was found to enhance rather than transform instructional practice in the physics classroom, despite its frequent use and positive outcomes. Further, students perceived the mobile devices as being very useful for their physics learning, and the frequency of their use was positively correlated with their perception of usefulness. In both the classroom and after-school, students’ interest and achievement in physics were positively influenced by the use of mobile technology. Despite its benefits in effectively presenting learning contents and enhancing students’ achievement (Jimoyiannis and Komis, 2001; Owen et al., 2008; Shieh et al., 2011; Shi and Liang, 2012), technology has the potential to make the study environment more complicated and more difficult to manage because technology brings distractions (Sailer and Hassenzahl, 2000; Pool et al., 2003a,b; Fried, 2008; Wurst et al., 2008; Kay and Lauricella, 2011).

Compared to other aspects of self-regulation, learners’ environment management, particularly management in technology-based physics learning environment, has been rarely investigated (Wolters, 2003; Du, 2016). More knowledge of factors that affect environment management in technology-based physics learning can help teachers make use of strategies or resources from motivational and self-regulated learning perspectives to decrease distractions brought by technology integration while maintaining its benefits. Hence, the purpose of the current study is to propose and examine a comprehensive model that uses motivational and self-regulated variables to explain factors affecting environment management in technology-based physics learning among Chinese secondary school students.

## 2. Literature review

### 2.1. Environment management in self-regulated learning

Self-regulated learning is “an active, constructive process whereby learners set goals for their learning and then attempt to monitor, regulate, and control their cognition, motivation, and behavior, guided and constrained by their goals and the contextual features in the environment” (Pintrich, 2000b, p. 453). Based on previous models of self-regulated learning (Winne and Hadwin, 1998; Zimmerman, 1998a,b, 2000), Pintrich (2000a,b) proposed a conceptual framework for self-regulated learning, where the regulation is composed of four perspectives, cognition, motivation/affect, behavior, and context, each having four phases, (a) forethought, planning and activation, (b) monitoring, (c) control, and (d) reaction and reflection. It has been tested in a variety of studies and the positive association between learners’ use of self-regulation strategies and their academic performances has been well demonstrated (Richardson et al., 2012; Broadbent and Poon, 2015).

Environment management, also known as environmental structuring (Thoresen and Mahoney, 1974; Zimmerman and Pons, 1986) or environmental control (Corno, 1993), appears in the reaction and reflection phase of both the behavior and context regulations in Pintrich’s (2000a,b) self-regulation model. It refers to learners’ behaviors of applying strategies to decrease distractions from the learning contexts by changing or adapting the environment to make it more conducive to task completion (Pintrich, 2000b; Wolters, 2003). Such attempts are a rather important aspect of self-regulation because all learning happens within certain learning contexts.

With the pedagogical shift from traditional teacher-centered classrooms where learners are passive recipients of teachers’ arrangements, to the popular student-centered classrooms where learners have more autonomy to regulate their studying, including the tasks, learning atmosphere and class structure, the skills in managing the environment are becoming particularly significant (Pintrich, 2004). Meanwhile, as learners grow older, they also gain more freedom and opportunities to manage their studying environment outside the class (Pintrich, 2000b). Researchers found that learners used a wide range of behavioral strategies to regulate their environment or contexts. Some examples include (1) finding more suitable or comfortable locations such as quiet places or personally preferred surroundings, (2) eliminating technology distractions such as turning down the volume of music, movies or TV in the room, (3) decreasing social distractions such as asking friends to be quiet, or (4) listening to light music to avoid distractions from non-removable noises and increase attentiveness (Wolters, 1998, 2003; Bigenho, 2011).

However, environment management can be affected by a variety of factors, one of which is goal-setting from a motivational perspective. To become self-regulated learners, individuals should be able to know how to set up proper goals, what are needed to attain the goals, and how to actually take actions to reach the goals (Dabbagh and Kitsantas, 2012). This implies the guiding position of goals in the planning stage, making that the purpose of using any self-regulated learning strategies, such as environment management, is to facilitate the goal reaching and learning task completion.

There are two types of goals. One is learning-oriented goal, which is the goal to do well with the learning (Xu et al., 2015). It has been found that when a learning goal is clearly set, students are more likely to be motivated to remove obstacles that would potentially prevent them from reaching the goals (Corno, 2001). Another type of goal is social-oriented, which is the goal to seek approval from teachers or peers (Xu et al., 2017a). When individual’s learning-related behaviors are approved and supported by those who are important to them, such as their teachers, peers and parents, it is more likely for these learners to take actions that can assist the completion of their learning activities, such as actively structuring their studying environment (Fishbein and Ajzen, 1975; Ajzen, 1991).

In addition to the influences of learning-oriented and social-oriented goals, time management, which involves making schedules for the learning tasks and allocating time for a variety of activities (Pintrich, 2000b), would also affect study environment management. One interesting point in the self-regulated model is that time management is always listed and measured together with study environment management, inferring some extent of inner links between these two (Pintrich, 2000b, 2004). This is not surprising because when a schedule is well-made, in order to stick to this plan, one needs to ensure the environment is supportive rather than detrimental to the carrying out of the planned schedule.

### 2.2. Environment management in technology-based learning

Research pertaining to self-regulation in technology-based learning has demonstrated the association between environment management and a variety of other regulative strategies. One example is Whipp and Chiarelli’s (2004) case study of six graduate students studying online courses. Through interviewing students and teachers as well as analyzing students’ reflective journals, Whipp and Chiarelli (2004) found environment management was a frequently used self-regulated learning strategy during their online learning; furthermore, these students’ successful management of their technical and social environment has largely shaped the impact from their motivation (goal orientation) to self-regulation. Besides, quantitative research found a positive relationship between environment management and time management. Specifically, it has been identified that groupwork time management was positively related to students’ efforts in arranging the study environment (Xu et al., 2013, 2017b).

Factors affecting students’ environment management in technology-based learning have also been investigated. For instance, Du et al. (2015) found social-oriented reasons, which mean that learning could bring opportunities to work with, learn from, and get support from peers, were a significant predictor of environment management at both individual and group levels; in contrast, learning-oriented reasons, which include obtaining skills and enhancing productivity, were not significantly related to environment management at any level. However, Du (2016) reached different findings in another study, showing that environment management was positively related to peer-oriented reasons at the individual level, and was positively related to learning-oriented reasons at the group level.

As discussed by these researchers, the significant positive relationship with peer-oriented reasons at the individual level might be a result of the fact that Chinese students do not want to let their group members down when working in a group due to their sense of accountability in a collective culture (Chiong and Jovanovic, 2012). The relationship between peer-oriented reasons and environment management was not significant because in contrast to the western value of learning from peers, Chinese tend to respect teachers and treat them as authority and major sources of knowledge. This makes the concept of learning from peers usually weak. Further, the emphasis of competition with peers in Chinese educational system is innately opposed to learning through collaboration, which would make Chinese learners’ peer-oriented goals less important at the individual level (Zhu et al., 2009). In addition, the value of hard work, personal improvement and academic progress in Confucian culture would make Chinese learners pay special attention to learning-oriented goals (Lee et al., 2003; Wang, 2004; Fang, 2007), which might not be applicable to students in United States.

### 2.3. Environment management in technology-based physics learning

Physics is one of the science subjects based on experiments, where the core is the understanding and mastery of abstract ideas and concepts (Chandra and Watters, 2012). Traditional physics classes which are often featured with chalk and talk are criticized by students as boring, irrelevant, less interactive and ineffective (Jimoyiannis and Komis, 2001; Williams et al., 2003; Angell et al., 2004). With the facilitation of technical equipment, however, teachers could have more flexible instruction and better draw on learners’ multi-sense when they are interacting with physics knowledge and activities presented by multimedia (Kenny et al., 2006; Shi and Liang, 2012). The use of animations that visualize the concepts would also make learners comprehend abstract concepts more effectively (Linn et al., 2006; Zucker and Hug, 2008) and facilitates students’ connection with prior knowledge and active engagement in reformulating their misconceptions (Jimoyiannis and Komis, 2001).

However, researchers and teachers have also found that the learning environment supported by technology becomes more complicated and have more distractions. Students are often multi-tasking when they have their laptops or mobile phones by their side--messaging, surfing the internet, playing games, listening to music, watching videos, or simply dealing with pop-up windows and low-battery or software update warnings while they are having classes or doing their homework (Hembrooke and Gay, 2003; Fried, 2008; Junco, 2012; Zhang, 2015; Chen and Yan, 2016). As individuals have limited mental resources to process information (Lang, 2000), potential distractions caused by technology equipment, especially that with Wi-Fi-access, would lead to attention shifts and cognitive overloads (Fried, 2008). When attentional demands go beyond the capacity to process information, learners’ performance will be negatively impacted (Posner, 1990; Sana et al., 2013). Such a negative association between learners’ multitasking and learning performance reveals the great need for students to self-regulate their learning through the successful management of the environment with technology devices (Zhang, 2015).

The introduction of technology into education has resulted in different means of utilizing technology for science education, for instance, to use mobile games (Avouris and Yiannoutsou, 2012; Nelson et al., 2017), specific apps (Szklanny et al., 2017; Buongiorno and Michelini, 2021), mobile technology to support collaborative learning (Tinker and Krajcik, 2012; Córdova Martínez and Alfonte Zapana, 2020; Telfer-Mason, 2021) or to enhance learning outcomes (Cahyana et al., 2019; Zhai et al., 2019). However, few studies were conducted to analyze the relationship between learning-oriented social-oriented goals, time management and environment management. Considering the role of physics in various professions and fields (Darma et al., 2019) and scarcity of relevant empirical studies about the aforementioned variables, there is an urgent need to investigate the factors affecting environment management by establishing and verifying a model addressing the relationships between motivational and self-regulated variables environment management in technology-based physics learning context.

### 2.4. The present study

The present study incorporated two important motivational variables (learning-oriented social-oriented goals) and two important behavioral self-regulation variables (environment and time management) to investigate the potential relationships among them, in the context of technology-based physics learning at Chinese secondary schools. More specifically, it is hypothesized that in line with previous research in other contexts, students’ environment management is positively associated with their learning-oriented goals, social-oriented goals and time management.

## 3. Materials and methods

### 3.1. Participants and procedure

Participants of the study were middle school students from one public middle school in south-eastern China. With permission from school superintendents, surveys were distributed by school principals and teachers, and consent forms were sent to students before they took the surveys. A total of 726 students (46.6% were male) from 51 classes participated in this study, with a response rate of 88.9%. Participants who failed to respond to the entire questionnaire were deleted. Since this study requires students to answer questions anonymously, each participant is not identifiable, so we could not get access to the belonged class information, we can only know how many students in total come from 51 classes, but we could not know which class each student came from.

All the students in the study received technology-based physics learning. They were taught physics utilizing online multimedia platforms. Teachers also made physics views more accessible by appropriate pedagogy. For example, teachers use multimedia simulation instead of artificial physics experiment, which can increase the flexibility of the scene. Using such technology-based learning approach can downplay the traditional teaching model that the teacher dominates the classroom. In addition, teachers will teach students according to their ability levels. Multimedia platforms will be used to set teaching content of different ability levels. Students can complete corresponding learning tasks according to their own ability level on the platform, so that they can gradually master the knowledge to be taught.

### 3.2. Measures

The development of the survey was informed by theory and research related to self-regulated learning and environment management. Several multi-item scales to measure each variable in the research model were used for this study (see Table 1).

**Table 1:** Results of confirmatory factor analysis.

	**Item**	**UE**	*t*-value***	**SE**	**CR** ^**b**^	**AVE** ^**c**^
Environment management (EM)	Q1	1.00	---^a^	0.54	0.75	0.51
	Q2	1.50	12.383	0.79		
	Q3	1.64	12.415	0.78		
Learning-oriented goal (LOG)	Q4	0.99	23.779	0.82	0.87	0.69
	Q5	1.02	24.674	0.85		
	Q6	1.00	---^a^	0.82		
Social-oriented goal (SOG)	Q7	1.00	---^a^	0.85	0.88	0.71
	Q8	1.05	26.561	0.86		
	Q9	0.91	24.813	0.81		
Time management (TM)	Q10	1.00	---^a^	0.83	0.75	0.51
	Q11	0.71	11.981	0.53		
	Q12	0.82	13.768	0.74		

***p < 0.001.

UE, Unstandardized Estimate; SE, Standardized Estimate.

a ---This value was fixed at 1.00 for model identification purposes.

b CR = (Σ λ)^2^ / (Σ λ)^2^ + (Σ(1–λ^2^)).

c AVE = (Σλ^2^)/(Σλ^2^) + (Σ(1–λ^2^)).

#### 3.2.1. Environment management

Environment management refers to leaners’ attempt to structure and manage their study environment (Pintrich et al., 1993; Xu, 2008). This variable was measured with three items using a five-point Likert scale (“1” for “never” and “5” for “routinely”). The development of these items was informed by previous research on self-regulation (Zimmerman and Martinez-Pons, 1990; Wolters, 2003). A sample item is, “Find a quiet area.” The internal consistency of these items is 0.75.

#### 3.2.2. Learning-oriented goal

Leaning-oriented goal refers to leaners’ purpose of learning arising from reinforcement of learning and the development of self-regulated attributes. This variable was measured with three items adopted from previous study on homework purpose scale (Xu, 2010) using a four-point Likert scale (“1″ for “strongly disagree” and “4″ for “strongly agree). A sample item was, “Learning and using computer helps me learn to work independently.” The internal consistency of these items was 0.87.

#### 3.2.3. Social-oriented goal

Social-oriented goal refers to learners’ purpose of learning influenced by teachers, parents, and peers. This variable was measured with three items adopted from previous study on homework purpose scale (Xu, 2010) using a four-point Likert scale (“1” for “strongly disagree” and “4” for “strongly agree). A sample item was, “Learning and using computer brings me teacher approval.” The internal consistency of these items was 0.88.

#### 3.2.4. Time management

Managing time refers to leaners’ attempts to plan, monitor, and regulate time use. Its development is informed by previous literature on time management in traditional classroom settings (Pintrich et al., 1993; Xu et al., 2014). This variable was measured with three items using a five-point Likert scale (“1” for “never” and “5” for “routinely”). A sample item was, “Set priority and plan ahead.” The internal consistency of these items was 0.75.

#### 3.2.5. Statistical analyzes

Structural equation modeling (SEM) was applied for data analysis in this study due to its advantage in simultaneously analyzing the relationships between latent and observed variables along with modeling random errors in the observed variables at a greater precision. The two-step approach to SEM (Schumacker and Lomax, 2010) was adopted. Confirmatory factor analysis (CFA) was performed first to estimate how well the observed variables measure the latent ones. In the second phase, the structural part of the SEM, the relationships among the exogenous and endogenous latent variables were estimated. To ensure the reliability in SEM, researchers (Kline, 2010) recommended a sample size of at least 100 to 150. Besides, Hoelter's (1983) critical N, which is the acceptable sample size for the proposed model at the.05 level of significance, was found to be 208 in the current study. Given that the sample size of this study is 726 (larger than 150 and 208), it should be adequate for the use of structural equation modeling.

## 4. Results

### 4.1. Descriptive statistics

Descriptive statistics, including the means, standard deviations, skewness, and kurtosis values for the 12 items in the survey were calculated. The means for those measured on a 5-point scale (i.e., Time management and Environment management) ranged from 2.22 to 3.55 and standard deviations ranged from 1.18 to 1.45. The values of the skewness and kurtosis for the 5-point items were from −0.43 to 0.85, and from-1.31 to 0.01, respectively. The variables measured on a 4-point scale (i.e., Learning-oriented goal and Social-oriented goal) had mean values ranging from 2.57 to 2.90 and standard deviations from.80 to 0.88. The values of the skewness and kurtosis for the 4-point items were from −0.57 to −0.02, and from −0.72 to.05, respectively. Since all values of the 12 items were considerably lower than the recommended cut-offs of|3.0|and|8.0|for skewness and kurtosis, univariate normality in the data was assumed (Kline, 2010).

### 4.2. Evaluation of the measurement model

A confirmatory factor analysis (CFA) was conducted with AMOS 24.0 using maximum likelihood estimation (MLE) and variance–covariance matrices to estimate a congeneric model with uncorrelated errors. As MLE is known to produce distorted results when the normality assumption is violated (Curran et al., 1996), multivariate normality was assessed using the Mardia measure of multivariate kurtosis (Mardia, 1970). The Mardia’s coefficient for the data in this study was 38.041, lower than the value of 168 computed based on the formula *p* (*p* + 2) where p equals the number of observed variables in the model (Raykov and Marcoulides, 2008). Accordingly, data in the current study applied the multivariate normality.

**Figure 1:**
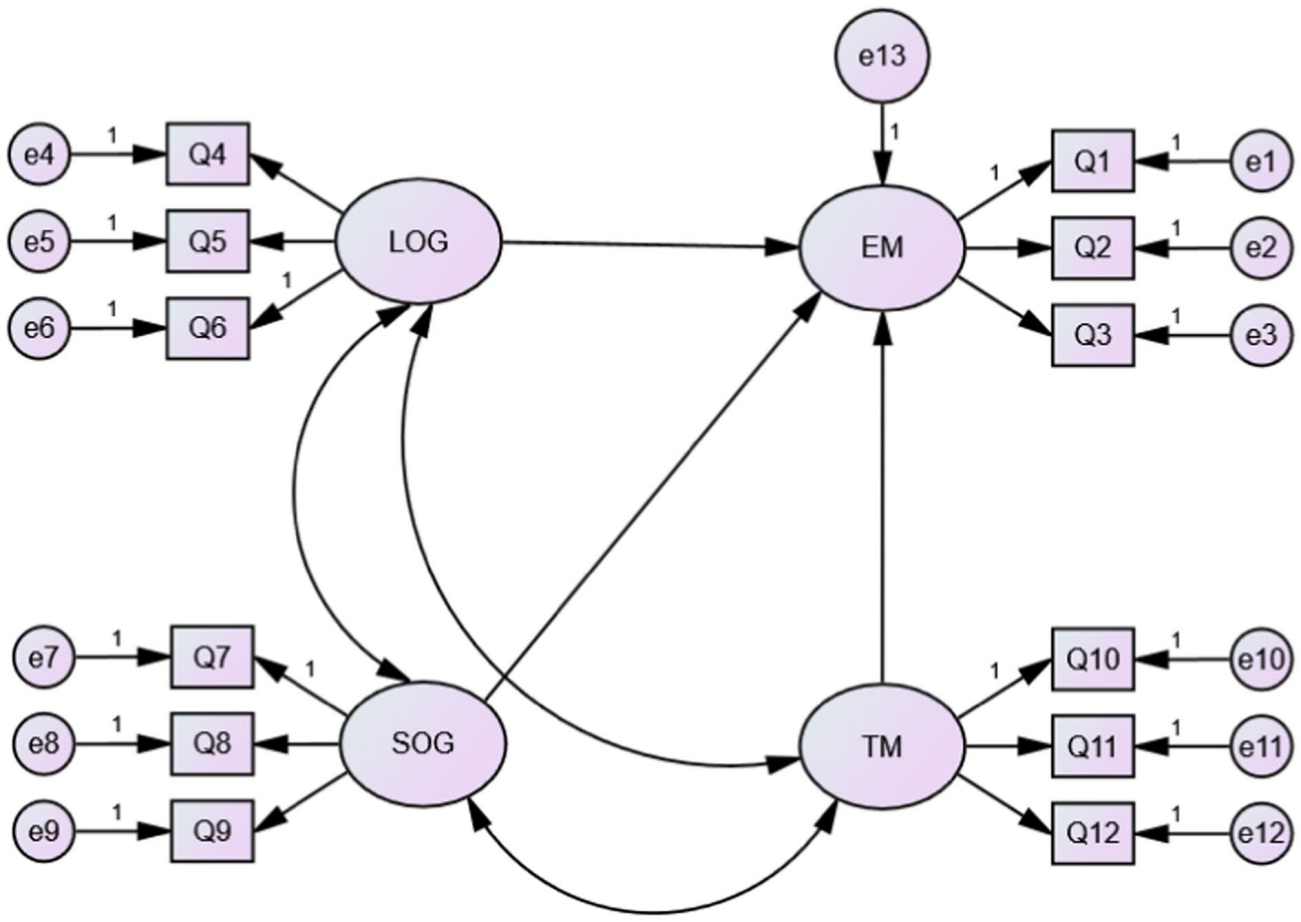
The research model.

The overall model fit was assessed using fit indices commonly reported in SEM studies, namely *χ*^2^, the ratio of *χ*^2^ to its degree of freedom (*χ*^2^/df), Tucker-Lewis index (TLI); comparative fit index (CFI); root mean square error of approximation (RMSEA); and standardized root mean square residual (SRMR). For chi square statistic, a good model fit would provide an insignificant result at a 0.05 threshold (Barrett, 2007). As the Chi-Square statistic (*χ*^2^) is a statistical significance test in nature, it is sensitive to sample size, meaning that the Chi-Square statistic tends to reject the model when large samples are used (Bentler and Bonnet, 1980; Jöreskog and Long, 1993). To minimize the impact of large sample size on the model in this research, Wheaton et al. (1977) recommended to use relative/normed chi-square (*χ*^2^/df). For the ratio of chi square to its degree of freedom, researchers have different thresholds for the indication of a good model. Recommendations for a good model fit range from 2.0 to 5.0 (Wheaton et al., 1977; Tabachnick and Fidell, 2007). Researchers suggested that values of TLI and CFI in the range of 0.90 through 0.94 may be considered as reasonable indicators of good model fit (Hair et al., 2010). According to Hu and Bentler (1999), a value that is greater than 0.95 is appropriate. The RMSEA with a value close to.06 (Hu and Bentler, 1999) or an upper limit of.07 (Steiger, 2007) tends to be agreed to have good fit, while a slightly larger acceptable range is between 0 and 0.08 is also acceptable (MacCallum et al., 1996; Hooper et al., 2008). The SRMR values between 0 and.1 is acceptable, with those less than.05 indicating well fit (Byrne, 1998; Diamantopoulos and Siguaw, 2000). Considering the large sample size of the model, despite the significant result of Chi square statistic, the measurement model revealed that the proposed model has a good fit to the sample data (*χ*^2^ = 227.841, *p* < 0.001; *χ*^2^/df = 4.747; TLI = 0.935; CFI = 0.953; RMSEA = 0.072; SRMR = 0.046). The testing results of the overall model fit are presented in Figure 1.

The reliability and validity of the items and the factors in the proposed model (see Figure 1) were assessed using the composite reliability (CR) and average variance extracted (AVE), respectively. Cronbach’s alpha was not reported in this study because when used with a multi-item scale, as is the case in this study, it was inclined to violating key assumptions (Teo and Fan, 2013). To assess the item validity, the direction, magnitude, and statistical significance of each parameter (*t*-value) were examined (Schumacker and Lomax, 2010). If the standardized estimate was greater than 0.50, it indicated that an item explained its latent variable well (Hair et al., 2010). Average variance extracted (AVE), which measures the amount of variance captured by the construct in relation to the amount of variance attributable to measurement error, was a more conservative indicator of validity and was computed for each construct. Both the CR and AVE are judged to be adequate when they equal or exceed 0.50 (i.e., when the amount of variance captured by the construct exceeds the variance due to measurement error) (Fornell and Larcker, 1981). The t-values, standardized estimates, CR, and AVE of all items and variables meet the recommended guidelines (Fornell and Larcker, 1981), as shown in Table 1.

### 4.3. Evaluation of the structural model

Evaluation of the structural model was made using the same model fit criteria for the measurement model. Results indicate that despite the significant chi square statistics, the structural model has a good fit (*χ*^2^ = 227.841, *p* < 0.001; *χ*^2^/df = 4.747; TLI = 0.935; CFI = 0.953; RMSEA = 0.072; SRMR = 0.046). 16% of the total variance in EM could be explained by LOG, SOG and TM in this model. As shown in Table 2, two out of three hypotheses in this study were supported by the data.

**Table 2:** Hypothesis testing results.

**Hypotheses**	**Path/correlation**	**Standardized path/correlation coefficient**	***t*-value**	**Results**
H1	LOG ➔ EM	0.406	6.015***	Supported
H2	SOG ➔ EM	−0.199	−3.237**	Not Supported
H3	TM ➔ EM	0.193	3.925***	Supported

*** *p* < 0.001; ** *p* < 0.01. LOG, Learning-oriented goal; EM, Environment management; SOG, Social-oriented goal; TM, Time management.

## 5. Discussion

The current study examined factors associated with study environment management in technology-based physics class at the secondary school level. Students’ learning-oriented goals and time management were revealed as positive predictors of environment management. Interestingly, social-oriented goals were found to be negatively associated with environment management. This paper has provided a deeper insight into the mechanism among the variables in technology-enhanced physics learning context in China, especially in light of the global COVID-19 pandemic, under which opportunities for implementing online education at a large scale have been provided (Xie et al., 2020). For learning in technology-enhanced context, one challenge faced by students is difficulty focusing during learning. Self-control from being distracted by various social media platforms, games, and apps available is essential to continue focusing on learning. In addition, in the current era of globalization, it is unavoidable that rapid development of information technology will have a profound impact on education. Educational stakeholders must continue to adapt technological developments to efforts to improve quality (Maruf et al., 2019).

Furthermore, the analysis of the relationship undertaken here, has extended our knowledge of the use of ICT in education, because the research focus is different from prior studies on how to use mobile games (Avouris and Yiannoutsou, 2012; Nelson et al., 2017), specific apps (Szklanny et al., 2017; Buongiorno and Michelini, 2021), mobile technology to support collaborative learning (Tinker and Krajcik, 2012; Córdova Martínez and Alfonte Zapana, 2020; Telfer-Mason, 2021) or to enhance learning outcomes (Cahyana et al., 2019; Zhai et al., 2019). The results provide a basis for future studies that may involve other crucial but less investigated variables in the context of technology-based learning environments.

Firstly, our study showed that learning-oriented goals had a positive influence on study environment management. This is consistent with findings in the study by Whipp and Chiarelli (2004) that students’ successful management of study environment was largely influenced by goal orientation. One possible explanation is that learning-oriented goals can help students regulate their learning process by choosing suitable strategies (Winne and Hadwin, 1998; Zimmerman, 1998a; Pintrich, 2000a,b, 2004) and setting up study environment is one of those strategies. In technology-based physics class, students with strong learning goal would pay close attention to the complicated knowledge content by removing things from the table to make enough space to do the calculation. For some individual learning activities such as watching teaching videos or doing virtual experiments, students would find a quiet place so that they can stay focus on the task. Another point to note is that Chinese students are well-known for their strong learning goals and eagerness for academic success (Xu et al., 2017b). Hence, the participants in our study might have stronger motivation to manage their learning environment due to their strong ambitions of achieving learning-oriented goals.

In addition, time management was found to affect environment management in technology-based physics class. This is in consistent with the findings in previous studies (Xu et al., 2013). It makes logical sense that in technology-based physics class, time management is a challenge to get used to technology to conduct different study activities while learning complicated physics knowledge at the same time. Therefore, those students who can effectively manage their time would try to eliminate possible distractions by setting up adequate study environment. External distraction such as family chatting and internal distraction such as students’ negative attitude when facing difficult problems would interfere and slow down learners’ study efficiency. Furthermore, scholars have also pointed out that technology itself might cause distractions because of multitasking behaviors of students when using technology (Junco, 2012; Zhang, 2015). When students have strong motivation to manage their time, they will try their best to ignore irrelevant time-consuming behaviors such as pop-up messages when using technology, to self-regulate internal distraction, and to remove external distractions which therefore lead to better environment management.

Interestingly, results in the present study indicate that social-oriented goals are negatively associated with environment management. This is probably because students who value social-oriented goals would care more about others’ opinions and approval. Their focus might transfer from learning-oriented reasons to social-oriented reasons and this might lead to learning in an environment which is not suitable for students’ individual preferences. As Sun (2017) points out in his study about social-oriented self, under the Confucian culture, Chinese individual would choose to listen to others’ advice and safeguard the interests of others when they were in conflict situations. Although they have the good intention to learn in the first place, they would be impacted by social diversity of others’ opinions and lose their independent critical thinking. Hence, in this scenario, social-oriented reasons might become distraction that is not good for environment management. Another possible explanation is that in an educational system that emphasizes competition (Zhu et al., 2009) and hard work (Li, 2005), learners may receive huge amount of pressure from their parents and teachers. Since adolescents can easily develop rebellious attitudes toward their parents’ and teachers’ high academic expectations (Luthar and Ansary, 2005), they might also go to the opposite direction by not regulating their learning to show their rebellion.

Two possible limitations that are needed to be addressed in our study. First of all, we only used cross-sectional data, which may not represent a causal relationship among the tested variables. Then, we only adopted a self-report survey in the study, which may be biased by one’s socially desirable bias.

## 6. Implications and recommendations

The findings of this study have important implications for teachers who use technology to teach sophisticated subject knowledge such as physics. Due to the complexity of such an environment, which is represented by high cognitive demands in content comprehension (Williams et al., 2003; Chandra and Watters, 2012) and great challenges in dealing with environmental distractions from technology (Kay and Lauricella, 2011), teachers’ efforts in improving learners’ motivation to learn knowledge and skills and at the same time decreasing their overly concern about other people’s opinions on them could help them better concentrate on learning itself and promote their management of studying environment. In the meantime, if the negative relationship between social-oriented goals and environment management comes from adolescents’ rebellion, teachers and parents should make changes so as not to overly put pressure on the students.

In addition, because learners have limited cognitive resources, when making teaching plans, teachers are recommended to take both the teaching content and mode (e.g., the use of technology) into consideration by weighing their challenging levels. For example, if the content is cognitively demanding, students’ attention would be more likely to be attracted by technology in their environment, which looks interesting with less cognitive loads (Fried, 2008). Accordingly, when the challenging content is part of the class goals, it would be helpful if teachers split it into a series of steps to teach to decrease the possibility of technological distractions.

Future studies may consider continuing this important line of research by incorporating other significant but less investigated variables (e.g., emotion management, help seeking) in the context of technology-based learning environments. Multiple data sources could also be drawn on to obtain a more comprehensive understanding of learners’ self-regulated learning. It would also be interesting to test if the relationships identified in this study could apply to other less challenging content courses supported by technology, so as to study whether the difficulty level of subject content could influence learners’ goals and further the relationships between goals and self-regulation strategies. Furthermore, it requires further investigation whether there are competitive relationships between variables that are associated with the same variable (e.g., learning-oriented goals and social-oriented goals in the current study). This will add up understanding about how to adjust different types of goals to enhance their joint positive effects on learners’ application of useful self-regulated learning strategies.

## Data availability statement

The original contributions presented in the study are included in the article/supplementary material, further inquiries can be directed to the corresponding author.

## Ethics statement

The studies involving human participants were reviewed and approved by the University of Macau, Information and Communication Technology Office. Written informed consent to participate in this study was provided by the participants’ legal guardian/next of kin.

## Author contributions

SX conceived of the presented idea and drafted the manuscript. YY developed the theory and verified the analytical methods. TC and TT performed the computations. All authors discussed the results and contributed to the final manuscript.

## Conflict of interest

The authors declare that the research was conducted in the absence of any commercial or financial relationships that could be construed as a potential conflict of interest.

## Publisher’s note

All claims expressed in this article are solely those of the authors and do not necessarily represent those of their affiliated organizations, or those of the publisher, the editors and the reviewers. Any product that may be evaluated in this article, or claim that may be made by its manufacturer, is not guaranteed or endorsed by the publisher.
